# Deficient Muscle Coordination Patterns of Reactive Stepping Responses in People With Chronic Stroke

**DOI:** 10.1177/15459683251369502

**Published:** 2025-09-15

**Authors:** Wouter Staring, Lotte van de Venis, Sarah Zandvliet, Digna de Kam, Teodoro Solis-Escalante, Alexander Geurts, Vivian Weerdesteyn

**Affiliations:** 1 Department of Rehabilitation, Donders Institute for Brain, Cognition and Behaviour, Radboud University Medical Center, Nijmegen, The Netherlands; 2 Department of Rehabilitation, Sint Maartenskliniek, Nijmegen, The Netherlands; 3 Erasmus MC, University Medical Center Rotterdam, Department of Rehabilitation Medicine; 4 Department of Research, Sint Maartenskliniek, Nijmegen, The Netherlands

**Keywords:** muscle synergy, reactive balance, stroke, stepping

## Abstract

**Background:**

People with stroke often have persistent balance impairments that have a profound impact on mobility and daily life independence. Several studies have been conducted to identify stroke-related deficits in neuromuscular responses to balance perturbations. Yet, the majority of these studies involved low-intensity, non-stepping perturbations, whereas falling typically occurs at high-intensity perturbations where stepping is a key saving strategy.

**Objective:**

We aimed to identify deficits in muscle coordination patterns of reactive stepping in people with supratentorial stroke (PwS).

**Methods:**

We included 32 PwS, who performed multidirectional stepping responses with their paretic and non-paretic leg. We determined step quality, and performed muscle synergy analysis to characterize stance- and swing-leg muscle coordination patterns.

**Results:**

We observed smaller leg angles in PwS in lateral, posterolateral and posterior directions, particularly with the paretic leg. Muscle synergy analysis yielded a set of 5 synergies in both groups for the swing VAF_Paretic_ = 0.84 ± 0.02, VAF_Non-Paretic_ = 0.84 ± 0.02) and stance leg VAF_Paretic_ = 0.85 ± 0.02, VAF_Non-Paretic_ = 0.84 ± 0.02). Three synergies were less frequently represented during paretic step execution. In addition, for the synergy with prominent gluteus medius involvement, underrepresentation was associated with lower Fugl-Meyer lower-extremity scores.

**Conclusions:**

The finding of deficient synergy structure and activation during reactive stepping complements and extends insights into balance related impairments after stroke. As the key next step, the methodology presented here allows identifying whether training-induced gains in reactive step quality are related to optimization of pre-existing coordination patterns, or whether some degree of behavioral restitution (i.e., return to “normal” coordination patterns) may still be possible.

## Introduction

People with stroke (PwS) often have persistent balance impairments, with recent work showing that this is even true for those who sustained a mild stroke.^1^ These balance impairments have a profound impact on mobility and daily-life independence, and are a key risk factor for falling.^2^ PwS show 2 (mild stroke) to 10 (severe stroke) times higher fall rates than their healthy counterparts, and a 4-fold greater odds of sustaining a hip fracture in the event of a fall.^1,3^

To gain insight into the apparent difficulties of PwS to recover from a loss of balance, several studies have been conducted to identify stroke-related deficits in neuromuscular responses to balance perturbations.^4-6^ These studies mainly focused on the automatic postural response (APR), which is considered the first line of defense against a loss of balance. APRs are characterized by stereotyped recruitment of a set of muscles (with onset latencies 100-200 ms post perturbation) to counteract the initial movement of the center of mass (CoM), and are presumably mediated by subcortical postural neural circuits.^7^ It was found that PwS have delayed muscle onset^5^ and reduced APR amplitude^6^ when confronted with a loss of balance. APR muscle coordination was more impaired in individuals who were unable to sustain the imposed perturbation compared to those who recovered successfully without stepping.^5^

A majority of these studies involved rather low-intensity perturbations that could be overcome using feet-in-place strategies. Yet, to prevent ourselves from falling following more challenging perturbations, reactive stepping is a key saving strategy. Recent work from our group has shown that stroke-related impairments in reactive balance capacity are more profound in stepping as compared to feet-in-place strategies.^5^ PwS have been reported to exhibit slower stepping responses and smaller step sizes, and to require more steps to recover balance compared to their healthy counterparts, particularly when stepping with the paretic leg.^8,9^

To elucidate the underlying neuromuscular control deficits, De Kam et al^4^ investigated whether defective APR recruitment (i.e., delayed onset and low amplitude) was associated with reactive stepping capacity in PwS. While some associations were found, a substantial degree of variance remained unaccounted for. In the same vein, training-induced improvements in reactive stepping capacity were found to be unrelated to changes in APR recruitment.^10^ As stepping to recover balance requires a coordinated response of task-specific stance- and swing-leg muscle recruitment that extends well beyond the APR time window,^11^ elucidating stroke-related neuromuscular control deficits of reactive stepping responses may yield important insights in fall mechanisms, and perhaps provide targets for preventive strategies.

In this study, we aimed to identify stroke-specific deficits in muscle coordination patterns of reactive stepping. We used muscle synergy analysis to identify deficits across multidirectional stepping responses in paretic and non-paretic swing and stance legs. Recent studies have suggested that this analysis yields a task-specific representation of neuromuscular coordination patterns,^12^ where the term “muscle synergy” refers to a coactive recruitment pattern of a group of muscles (not to be confused with pathological synergies). Muscle synergies have been used to evaluate neuromuscular coordination patterns in a variety of movements in healthy individuals and in participants with neurological conditions.^4,13-19^ In PwS, the number of synergies when performing a movement has often found to be lower compared to healthy counterparts,^17,18^ which was found to be associated with poorer scores on clinical scales of motor recovery.^4,19^ We therefore hypothesized that, compared to healthy individuals, PwS would have a lower number of muscle synergies in the paretic swing and stance leg, whereas the number of non-paretic leg synergies was expected to be the same. In addition, we determined whether the anticipated deficits in muscle coordination patterns in PwS were associated with clinical leg motor recovery and with reactive step quality.

## Methods

### Participants

We included 32 participants beyond 6 months after a unilateral supratentorial stroke and 14 healthy controls in the same age range (Table 1). The individuals with stroke were participants of the ROADS study, an open-label randomized control trial assessing the effects of perturbation-based training in chronic stroke (NL7730). We used their baseline data for addressing the present research question. Inclusion criteria were an ability to walk and stand independently for at least ten minutes (Functional Ambulation categories > 3).^20^ Exclusion criteria included conditions in which physical exercise is contra-indicated; receiving physiotherapy focusing on balance that could not be stopped temporarily; previously having received perturbation-based training that included mechanical and/or visual perturbations; other musculoskeletal or neurological conditions affecting balance; orthopedic problems; persistent visuo-spatial neglect (Star-Cancellation Test ≤ 50)^21^; medication negatively affecting balance; behavioral or cognitive impairments that interfere with complying to the study protocol; pregnancy; and inability to give informed consent. All participants provided written informed consent. Experimental procedures were approved by the Research Ethics Committee of the Radboud University Medical Center (Nijmegen, The Netherlands, NL67690.091.18).

**Table 1.** Participant Characteristics.

	Stroke	Healthy controls
Age (years)	60 (12)	64 (8)
Sex (M/F)	25/7	12/2
MoCa	26(2)	27 (2)
Months since stroke	56 (6-188)	
Affected body side (R/L)	14/18	
MI-LE (%)	77 (19)	
FMA-LE (range: 0-28)	22.2 (4)	
FAC (4/5)	8/24	
TIS (range: 0-23)	15 (13-19)	
MiniBESTest (range: 0-28)	20 (17-24)	27 (27-27)
Vibration threshold at medial malleolus (range 0-8)	5.1 (2.3)	
Falls past year	0.7(0-7)	

*Note*. Mean (SD) are shown for Age, MoCa (Montreal Cognitive Assessment), Months since Stroke, Motricity-Index Lower Extremity (MI-LE), Fugl-Meyer Assessment Lower Extremity (FMA-LE) and Vibration threshold). Frequency is shown for Sex (Male/Female), Affected body side (Right/Left), and Functional Ambulation Category (FAC). Median and IQR are presented for Trunk Impairment Scale (TIS), Mini Balance Evaluation Systems Test (Mini-BESTest) and Self-reported falls in the past year (Mean & Range).

### Experimental Procedure

First, PwS came to the lab for an intake visit. During this visit, we obtained informed consent and conducted a number of clinical assessments, including Fugl-Meyer Assessment Lower Extremity (FMA-LE),^22^ Motricity Index (MI-LE),^23^ Trunk Impairment Scale (TIS),^24^ Mini Balance Evaluation Systems Test (Mini-BESTest),^25^ and vibration perception was assessed using a Rydel-Seiffer^26^ tuning fork at the medial malleolus. We also recorded self-reported number of falls in the previous year. In addition, we performed a familiarization session on the Radboud Fall Simulator (RFS) to determine whether participants could perform reactive paretic steps at the intended perturbation intensity. Within approximately 2 weeks after the intake, they visited the lab for a second time, which involved the reactive stepping assessment as further explained below. Healthy participants performed the MiniBESTest and reactive stepping assessment during 1 visit.

### Reactive Stepping Assessment

Reactive stepping was assessed on the RFS.^27^ Participants stood on the RFS without shoes or assistive orthoses with their feet 5 cm apart wearing a supporting soft ankle brace to prevent ankle sprains of the paretic leg. The RFS triggered reactive stepping responses by unexpected support-surface translations at an intensity of 1.5 m/s^2^ (300 ms acceleration; followed by 500 ms constant platform velocity, and 300 ms deceleration). Participants received specific instruction regarding which leg to use for stepping. The instruction was: “If you feel the need to take a step to regain balance, please do so with the *instructed leg.”* This instruction was used to enforce taking side steps (and avoid cross steps) in any lateral perturbation direction with both the paretic and non-paretic leg. Perturbation consisted of platform translations in posterior (POST), 45° posterolateral (PostLat), lateral (LAT), 45° anterolateral (AntLat), and anterior (ANT) directions. A brief familiarization was provided prior to the actual experiment, involving platform translations in all directions of increasing intensity up to 1.5 m/s^2^. Healthy participants performed approximately 20 practice trials in total, while stroke participants were exposed to approximately 20 trials per leg (i.e., 40 trials in total). The experimental protocol consisted of 8 series of 25 reactive stepping trials, each with 5 repetitions per direction, administered in random order. They performed 4 series with each stepping leg, in a randomly assigned order (Appendix 2). We set a variable inter-trial interval in the platform software ranging between 1 and 3 seconds. Participant were given resting breaks in between series of perturbations.

### Data Processing & Analysis

Ground reaction forces were recorded using 2 force plates embedded in the platform located under each foot (AMTI Custom 6 axis composite force platform, USA; size: 60 × 180 cm each; sampling rate: 2000Hz). In order to quantify stepping characteristics, 3D kinematics were recorded with an 8-camera motion capture system (Full Body Plug-in Gait model excluding head and arm markers; Vicon motion systems, United Kingdom, 100 Hz). Electromyographic data (EMG; Cometa, Italy) were collected bilaterally from erector spinae (ERSP), gluteus medius (GLUT), biceps femoris (BFEM), semitendinosus (SEMT), rectus femoris (RFEM), peroneus longus (PER), tibialis anterior (TA), and soleus (SOL) at 2000 Hz. All electrodes were placed according to SENIAM guidelines^28^ except for the soleus muscle, which was placed toward the lateral side of the leg to avoid artifacts caused by the narrow stance position.

### Spatiotemporal characteristics and step quality

In order to evaluate stepping behavior, we determined the spatiotemporal characteristics: step onset, step duration, and step length. To determine these characteristics, force plate data was first low-pass filtered at 80 Hz (5th order Butterworth IIR filters, zero-phase shift) to remove platform motor noise. Step onset was defined as the first moment when the vertical ground reaction force (GRF) surpassed a threshold of 20 N and verified by an increase in vertical velocity of the foot greater than 0.1 m/s. Foot strike was determined as the first moment when the selected force plate was (re)loaded with at least 50 N. Step quality was expressed by the angle of the stepping leg at the instant of foot landing.^29^ The leg angle was quantified as the angle between the vertical and a line connecting the mid pelvis and the mid foot position. Mid foot position was defined as the midpoint between the calcaneus and the 2nd metatarsal marker in the respective perturbation direction of the instructed leg.

### EMG Pre-processing

Individual EMG data of trials performed according to instruction (i.e., steps performed with the intended leg) was band-pass filtered (20-450 Hz), rectified and low-pass filtered at 40 Hz). EMG activity of each individual trial was time normalized according to perturbation onset and stepping events to account for temporal differences in muscle recruitment as described in a previous study by Staring et al^11^ Perturbation onset was set at 0 %, step onset at 50%, and foot strike at 100%. For each leg EMG data was concatenated into 8 (number of muscles) by n (number of trials) matrices and scaled to unit variance across all trials for equal weighting in the muscle synergy extraction.^30^ To account for differences in the number of stepping trials performed according to instruction between perturbation directions, we selected the number of trials concatenated across directions to be the lowest number of trials present in 1 direction, with a minimal threshold of 5 randomly selected trials.

### Muscle synergy analysis

We performed nonnegative matrix factorization (NNMF) to extract muscle synergies for each leg (paretic/dominant, non-paretic/non-dominant) and behavior (swing or stance). NNMF is a decomposition algorithm extensively used to characterize muscle recruitments,^12^ using the formula:

$$M_{m*n}=W_{m*s}*C_{s*n}+e,$$

where the EMG matrix M (muscles*trials) is decomposed into time-invariant structures W (number of synergies) and time varying coefficients C (samples) + e, the residual error matrix. The goodness of fit of the reconstructed structures and coefficients was reflected by the variance accounted for (VAF). We determined the minimal number of synergies that could account for >80% of the total variability computed as follows:

$$VAF=\left(1-\frac{var\left(M-W*C\right)}{var\left(M\right)}\right)*100.^{31}$$

To allow for comparison of synergies between stroke and healthy individuals, synergy structures were normalized by the maximum of each structure and corresponding time-varying activation coefficients were scaled by the same quantity.^13^

In the presently-used paradigm, healthy older individuals recruited 5 synergies in the swing and 5 synergies in the stance leg, irrespective of leg dominance.^11^ From these previously reported data, we generated a healthy reference set of synergies by pooling synergies from healthy individuals of both legs. Subsequently, to compare similarity of the time-invariant structures (W) between healthy individuals and PwS, we determined Pearson correlations between healthy reference set and individual synergies from PwS. Synergies were considered similar when r-values surpassed 0.707 based on an alpha level of 0.05 and 8 muscles included per synergy.^32^

### Statistical analysis

To identify differences in step characteristics between PwS and healthy controls, we performed linear mixed model (LMM) analyses with fixed factors *group* (healthy vs. stroke), *leg* (paretic or dominant vs. non-paretic or non-dominant), *stepping direction* (5 levels) and the respective interactions between these parameters.

The proportion of synergies similar to the healthy reference was compared across healthy, paretic and non-paretic legs using a Chi-squared test. The time-varying coefficients of synergies identified as similar were compared between the legs with a 1-way ANOVA (paretic/non-paretic/healthy) using statistical parametric mapping(SPM) with an α level of 0.05.^33^ Differences in time-varying activation of the respective synergies between legs were expressed as a percentage of time between perturbation onset and foot strike. All SPM analysis were performed within MATLAB R2023 using the spm1d.04 toolkit.^33^

In addition, we evaluated whether deficits in synergy structure (i.e., when a healthy reference synergy could not be identified) in PwS were related to leg motor recovery (FMA-LE scores), to reactive step quality, and to falls. To this aim, we compared the FMA-LE score between individuals in whom the respective muscle synergy could be identified to those who did not express this synergy using Mann-Whitney U tests. For falls, we performed a Chi-square test to examine the proportion of fallers (yes/no) and synergy presence (yes/no). In addition, we compared leg angles of stepping directions in which the respective synergy was predominantly active.

## Results

Of the 32 participants who were included, 2 participants were unable to complete study procedures during the reactive stepping assessment visit due to fatigue. Their data were not used in the analysis. In addition, 4 participants were unable to perform reactive steps with the paretic leg in the intake session (FMA-LE scores: 24, 20, 17, and 16). These individuals only performed non-paretic steps during the actual experiment. Hence, data analysis of spatiotemporal characteristics was performed on 26 participants with paretic steps and 30 participants with non-paretic steps.

All healthy participants were able to complete the experimental procedure and they performed 192 ± 8 steps according to instruction. PwS performed on average 80 ± 24 trials according to instruction with the non-paretic leg. One participant (FMA-LE: 13) only performed a single sequence of 25 non-paretic steps. A total of 24 PwS completed the full protocol with the paretic leg (100 trials) and performed on average 80 ± 25 trials according to instruction. Two individuals (FMA-LE: 11 and 23) performed only 2 series of trials with the paretic leg, as they were hardly able to take reactive paretic steps across directions (fewer than 5 “valid” trials except for the anterior direction), yet their trials according to instruction were used in the spatiotemporal analysis.

When instructed to step with the paretic leg, PwS experienced the least difficulties complying with the instruction in the anterior direction (median = 20, IQR = 20-20) and the most difficulties in the posterolateral direction (median = 16, IQR = 9-18). Similarly, when performing non-paretic steps, PwS experienced least difficulty in the anterior direction (median = 20, IQR = 18-20) and most difficulty in the posterolateral direction (median = 18, IQR = 8-19). Unsuccessful trials involved reactive steps where the initial step was performed with the non-instructed leg rather than instructed leg. None of the participants fell into the safety harness in any of the trials.

### Step quality and apatiotemporal characteristics

The results for the step quality and spatiotemporal variables are summarized in Table 2. The LMM yielded significant effects of group and leg as well as a significant group*leg interaction across spatiotemporal outcome measures. A full overview of the statistics can be found in Appendix 1. For paretic steps in PwS, we observed smaller leg angles (on average 1.6-2.0⁰) in lateral, posterolateral and posterior directions as compared to non-paretic steps (*P* ≤ .01), and by 1.7-4.5⁰ compared to steps of healthy participants (*P* ≤ .02). In addition, leg angles of non-paretic posterolateral and posterior steps were also 2.1-3.0⁰ smaller compared to the healthy participants (*P* < .01). Similarly, we observed 3.8-7.1cm smaller paretic and non-paretic step lengths in posterolateral and posterior directions compared to the healthy participants (*P* ≤ .05). Step duration was 24-41 ms shorter in non-paretic compared to paretic steps in posterior, posterolateral and anterior directions (*P* ≤ .01), and also shorter by 22-40 ms compared to healthy participants’ steps in these directions (*P* ≤ .02). We did not observe any significant differences in step onset latencies.

**Table 2.** Mean (SD) Step Quality and spatiotemporal step variables.

	Stepping leg	Post	PostLat	Lat	AntLat	Ant
Leg angle						
Stroke	Paretic	8.9 (4.3)*,**	12.0 (2.7)*,**	16.5 (2.8)*,**	16.6 (3.6)**	13.9 (3.0)**
	Non-paretic	11.0 (2.6)*	13.7 (2.4)*	18.1 (2.4)	18.2 (2.8)	15.5 (2.6)
Healthy	Dom	13.5 (1.9)	15.6 (2.7)	18.2 (2.3)	17.5 (2.4)	15.0 (2.0)
	Non dominant	13.8 (1.9)	15.9 (1.8)	18.2 (1.5)	17.5 (2.3)	15.0 (2.2)
Step onset (ms)						
Stroke	Paretic	340 (29)	401 (38)	427 (38)	414 (37)	426 (107)
	Non-paretic	349 (37)	400 (41)	424 (38)	418 (61)	410 (58)
Healthy	Dominant	344 (20)	393 (20)	420 (23)	408 (35)	419 (47)
	Non dominant	329 (22)	384 (34)	416 (29)	410 (38)	430 (40)
Step duration (ms)						
Stroke	Paretic	276 (66)	213 (46)	184 (32)	203 (42)	275 (61)
	Non-paretic	235 (40)*,**	187 (26)*,**	178 (19)*	193 (26)	233 (43)*,**
Healthy	Dominant	264 (37)	203 (30)	199 (22)	198 (23)	264 (35)
	Non dominant	293 (32)	213 (30)	184 (22)	200 (25)	255 (40)
Step length (mm)						
Stroke	Paretic	347 (118)*	363 (92)*	375 (73)	385 (78)	408 (81)
	Non-paretic	372 (86)*	370 (77)*	391 (63)	344 (84)	406 (75)
Healthy	Dominant	410 (54)	406 (68)	383 (56)	384 (47)	427 (44)
	Non dominant	437 (43)	422 (63)	413 (44)	397 (57)	430 (54)

* Significant difference between PwS and healthy participants.

** Significant difference between steps with paretic versus non-paretic leg.

### Muscle coordination patterns

Twenty PwS performed a minimum of 5 paretic steps in all directions and could be included in the paretic-leg muscle synergy analysis. Out of 30 PwS, 28 performed sufficient steps with the non-paretic leg. The 2 individuals who did not perform sufficient non-paretic steps were the abovementioned individual with a single sequence of 25 non-paretic steps and an individual who was not able to perform sufficient posterolateral steps. The average number of trials per stepping direction included in the synergy analysis was 15 (range 9-20) for the paretic leg and 15 (range 7-20) for the non-paretic leg.

**Figure 1.**
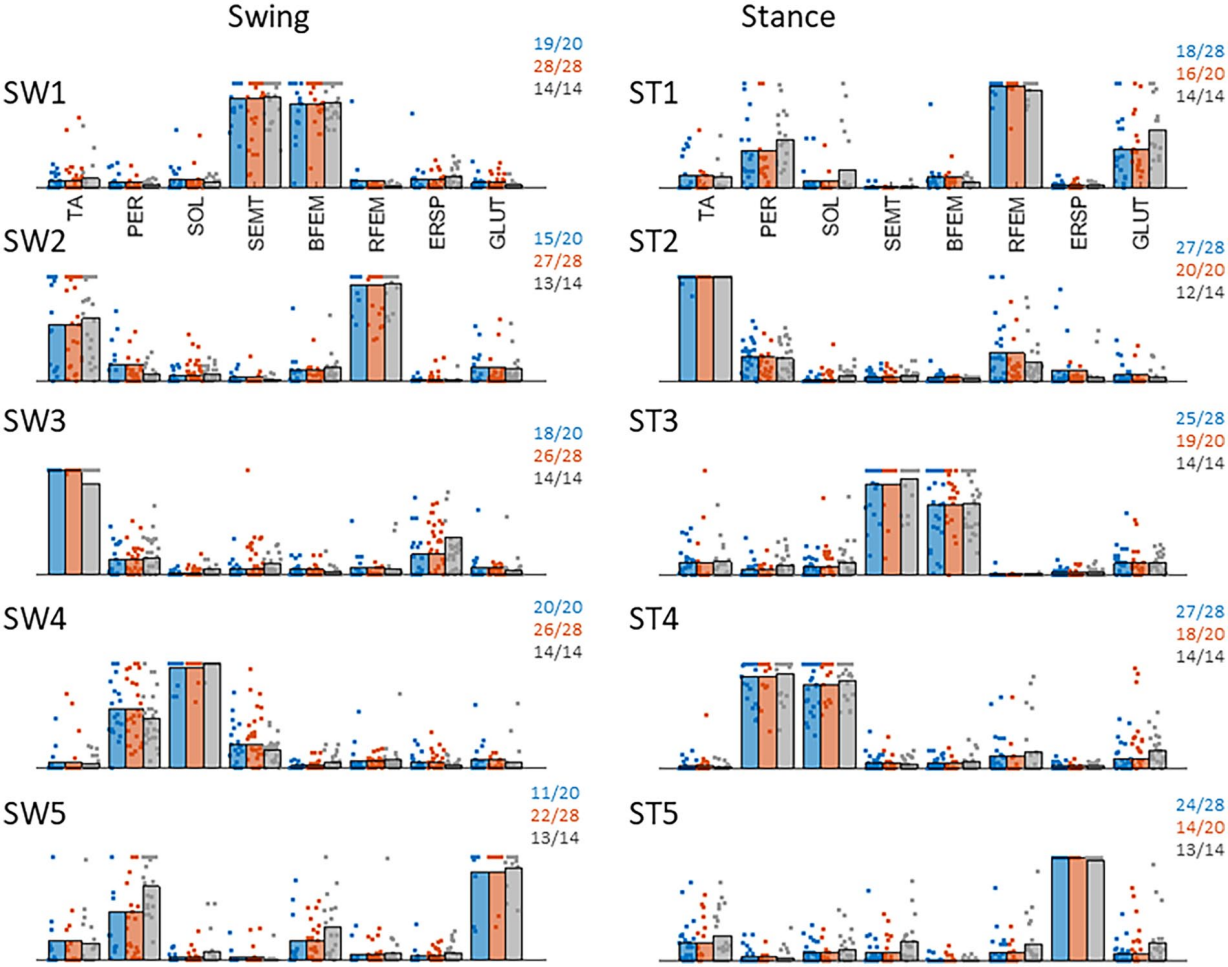
Representation of muscle synergies across groups. Left column shows swing leg synergies (rows 1-5) and right column reflects stance leg synergies (rows 1-5). Blue represents paretic leg synergies, orange represents non-paretic leg synergies and grey represents healthy reference leg synergies. Individual dots represent contributions of muscles across individuals. Note that individual dots are “stacked” horizontally when multiple individuals exhibit the maximum muscle contribution in the respective synergy. The numbers in the top right corner of each subplot reflect the number of participants out of the total group in whom the respective synergy was identified.

Similar to the results previously reported for the healthy participants,^11^ muscle synergy analysis in PwS yielded 5 synergies on average for the swing leg (average VAF_Paretic_ = 0.84 ± 0.02, VAF_Non-Paretic_ = 0.84 ± 0.02) and the stance leg (average VAF_Paretic_ = 0.85 ± 0.02, VAF_Non-Paretic_ = 0.84 ± 0.02) (Figure 1). Yet, comparison to our healthy reference set indicated significant differences in the structure of the extracted synergies (see numbers in Figure 1). Specifically, we observed that paretic swing-leg synergy SW2 (mainly involving TA and RFEM) was less frequently represented (15/20, X^2^ = 7.9, *P* < .01). Paretic swing-leg synergy SW5 (mainly involving GLUT and PER) was also less frequently represented (11/20, X^2^ = 5.7, *P* = .02). On the non-paretic side, we observed a similar lower representation for stance-leg synergy ST5 (mainly involving ERSP; 14/20, X^2^ = 5.8, *P* = .02).

Within our group of 20 paretic steppers, FMA-LE scores were significantly higher in participants with SW5 (median = 24) as compared to those without (median = 22, U = 22, Z = −2.1, *P* = .03), whereas MI, Mini-BESTest or TIS scores did not differ (all *P* > .33). No differences were observed in step quality (i.e., leg angles) between PwS with and without SW5 (U = 33, z = −.95 *P* = .34). For paretic-leg synergy SW2 and non-paretic leg synergy ST5, clinical characteristics or leg angles were not significantly different between PwS with and without the respective synergy. Yet, we found that 4/5 participants without synergy SW2 had fallen in the previous year versus 3/15 participants with SW2 (*P* = .015). No such differences were observed for synergies SW5 and ST5.

### Muscle synergy activation patterns

**Figure 2.**
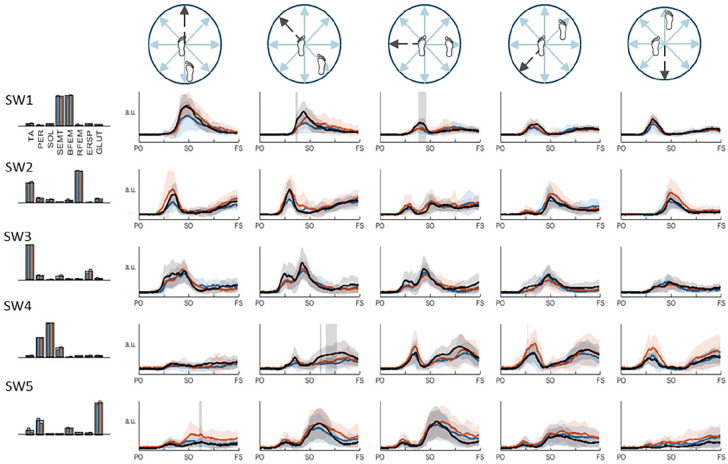
Swing leg muscle synergies and their activation coefficients per stepping direction. Mean activation (+SD) coefficients of the paretic leg are shown in blue, the non-paretic leg is shown in orange, and the healthy reference pattern is shown in black. Vertically orientated grey areas indicate a significant main effect of group (healthy vs. stroke) for each stepping condition. Abbreviations: PO, perturbation onset; SO, step onset; FS, foot strike.

In the swing leg, we observed largely similar direction- and time-dependent synergy activation patterns in both the paretic and non-paretic legs of PwS as compared to the healthy reference pattern, with a few significant between-group differences (Figure 2). Synergy SW1 (SEMT and BFEM) showed a bilateral underrecruitment in PwS shortly before step onset in the lateral and, to a lesser extent, posterolateral directions. During the swing phase, activation coefficients of SW4 (PER and SOL) were lower bilaterally in PwS in the posterolateral direction. In addition, non-paretic SW5 (GLUT and PER) recruitment in the posterior direction was higher compared to the healthy reference during the swing phase, albeit significant only for a brief epoch shortly after step onset.

**Figure 3.**
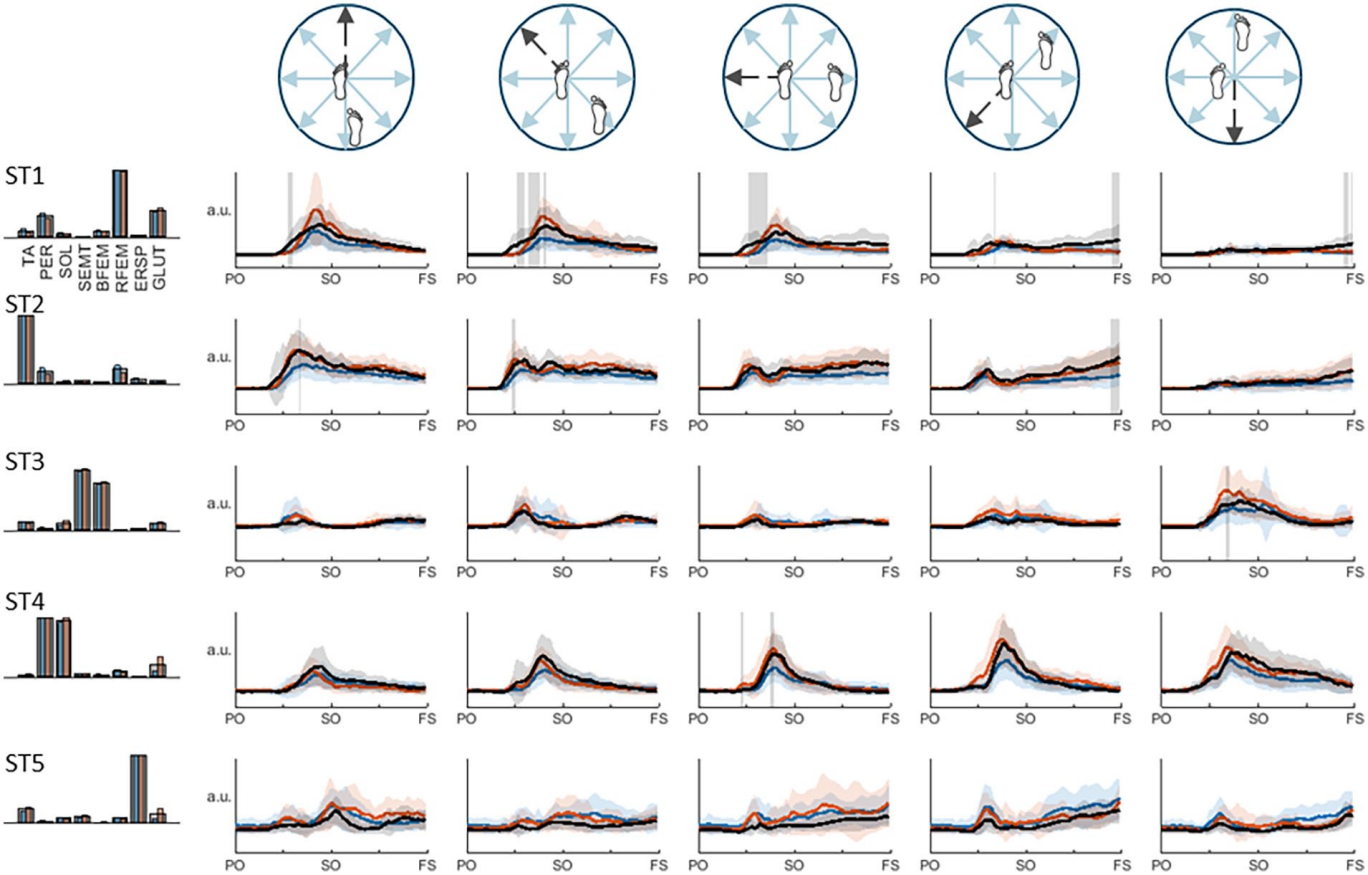
Stance leg muscle synergies and their activation coefficients per stepping direction. Mean activation (+SD) coefficients of the paretic leg are shown in blue, the non-paretic leg is shown in orange, and the average pattern of the healthy leg is shown in black. Vertically orientated grey areas indicate a significant main effect of group (healthy vs stroke) for each stepping condition. Abbreviations: PO, perturbation onset; SO, step onset; FS, foot strike.

Stance leg synergies of both the paretic and non-paretic leg also demonstrated largely similar activation patterns compared to the healthy reference pattern, with some significant differences (Figure 3). Prior to step onset, underrecruitment of paretic-leg synergies ST1 (RFEM, GLUT, and PER) and ST2 (TA) was observed in posterior and posterolateral directions, and ST1 and ST4 (PER and SOL) in the lateral direction. In the anterior direction, activation coefficients of non-paretic ST3 (SEMT and BFEM) were higher than the healthy reference pattern in this time window. In addition, prior to foot strike (of the contralateral swing leg) we found lower activation coefficients of paretic and non-paretic ST1 in the anterior and anterolateral directions, as well as paretic stance-leg ST2 in the anterolateral direction.

## Discussion

This study provides a first account of stroke-related deficits in the neuromuscular control of reactive stepping following multidirectional balance perturbations. First, we observed that step quality (i.e., leg angle) was poorer in PwS in lateral, posterolateral and posterior directions, particularly when stepping with the paretic leg. Our analysis yielded a similar set of 5 muscle synergies in healthy individuals and PwS for the swing as well as stance leg yet, we found that 3 of these synergies (SW2, SW5, and ST5) were less frequently represented during paretic steps in PwS. Moreover, SW5 was less frequently identified in participants with lower FMA-LE scores.

### Prominent Deficits in Synergy Structure and Activation During Stepping with the Paretic Leg

The finding that, during steps with the paretic leg, a number of synergies (SW2, SW5, and ST5) were less frequently identified in PwS is consistent with previous studies reporting “simplified” synergy structures across motor tasks.^15,18,34^ The most prominent deficit that we observed was in paretic swing leg SW5, which involves a prominent contribution of gluteus medius. Hence, it appears to play a key role in hip abduction for rapidly shifting weight toward the stance leg followed by quickly moving the stepping leg into the direction of the impending fall. The finding that PwS with lower FMA-LE scores were less likely to express this “hip abduction” synergy may be caused by pathological hip extension-adduction coupling, commonly observed after stroke.^35,36^ Concurrent recruitment of SW5 and “hip extension synergy” SW1 occurs around step onset, particularly in the posterolateral direction. Therefore, in PwS with poorer leg motor recovery, the recruitment of SW1 may have impeded the expression of the hip abduction synergy SW5. While the deficits were most pronounced in SW5, the significantly lower activation coefficients of SW1 around step onset suggest that the reverse may also have been true, albeit to a much lesser extent.

A lack of SW5 expression did not result in consistently poorer step quality, but closer inspection of individual results yielded a potentially interesting pattern. All participants *with* synergy SW5 consistently had high Fugl-Meyer scores (≥23 points) and comparatively good step quality (range 10-16⁰), whereas those *without* SW5, with in general lower Fugl-Meyer scores demonstrated more variable step quality (range 3-14⁰), with gluteus medius activity being recruited in diverse subject-specific. This might reflect recruitment of behavioral compensation strategies, apparently with a varying degree of successfulness in achieving proper stepping performance.

SW2 was another swing-leg synergy that was less frequently identified during paretic steps in PwS. In the posterior and posterolateral directions, where PwS demonstrated the greatest difficulties performing instructed paretic steps, SW2 is particularly active during the initial (APR) phase of the recovery response. This finding concurs with previous reports of discoordinated APR recruitment of tibialis anterior and rectus femoris during feet-in-place responses in PwS.^4,6^ Participants in whom SW2 was not identified were more likely to have fallen in the previous year (cf. those with SW2). While this result needs to be interpreted with caution due to the small group sizes, it appears to be in agreement with previous studies reporting poorer recovery capacity following backward perturbations associated with defective APR recruitment.^5,6^

While the poorer paretic step quality in the posterior, posterolateral and lateral directions was not univariately associated with missing synergies SW2 or SW5, it can be inferred from Figure 2 that reduced activation of paretic “hip extension” synergy SW1 (around step onset) and “ankle plantarflexion” synergy SW4 (during swing) also clusters in these stepping directions. The reduced activation of these synergies likely led to smaller paretic step lengths. We therefore suggest that a combination of deficits in the first 4 swing-leg synergies, rather than any one of these in isolation, may have contributed to the poorer paretic step quality in these directions.

When stepping with the paretic leg, we also found less frequent expression of synergy ST5 on the non-paretic stance side. As ST5 mainly involves the ERSP and is active across all directions, it likely accommodates trunk stability.^37^ This deficient recruitment at the non-paretic side may be related to axial muscles being (at least partly) innervated by uncrossed fibers of the corticospinal tract. Interestingly, no differences were observed in ST5 on the paretic stance side, suggesting that trunk stability during a non-paretic step may benefit from ipsilateral innervation from the non-lesioned hemisphere.^38^

### Stroke-Related Changes in Paretic and Non-Paretic Step Characteristics

Despite the observed deficits of APR-related recruitment, PwS demonstrated similar step initiation times compared to healthy controls. Step lengths, however, were smaller for both paretic and non-paretic leg, particularly in posterior and posterolateral directions. These observations are in agreement with previous findings, yet most of these studies did not differentiate between stepping legs.^5,39^ Two studies that did compare (instructed) paretic and non-paretic step characteristics following backward perturbations reported poorer stability at touchdown and lower multiple stepping thresholds for paretic.^9,40^ We confirm and extend these findings by showing poorer paretic step quality in all directions.

Steps taken with the non-paretic leg in the posterior and posterolateral directions were shorter in length and duration compared to the healthy controls, resulting in significantly poorer step quality. These stroke-related deficits in step characteristics may be due to the slightly lower activation of paretic stance-leg synergies ST1 and ST2 in these directions during the APR phase and the initial phase of the stepping movement. As these synergies contain key contributions of tibialis anterior and rectus femoris, their lower activation in the paretic stance leg likely results in smaller corrective torques and reduced braking of the backward CoM acceleration. This may leave less time for the non-paretic swing leg to “catch” the CoM, resulting in smaller leg angles. These observations are in line with previous findings of poorer paretic limb support and earlier fall onset when stepping with the non-paretic versus the paretic leg,^41^ and they extend previous findings on the impact of deficient paretic TA-RF recruitment in feet-in-place balance recovery.^4,6^

### Limitations and Implications

Our extensive reactive stepping protocol restricted the inclusion to a population of relatively well recovered PwS, which limits the generalizability of the findings to more severely affected individuals. Furthermore, in order to use synergy analysis for comparing muscle coordination patterns between the paretic and non-paretic legs in PwS and between PwS and a healthy reference group, we had to carefully select a single perturbation intensity that was feasible for a majority of PwS in the most difficult conditions (i.e., paretic posterolateral steps), yet just high enough to evoke consistent reactive stepping behavior in healthy participants. While the selected intensity of 1.5 m/s^2^ fulfilled the latter criterion, the more impaired PwS experienced substantial difficulties taking reactive steps with the paretic leg. Only 20 out of the 32 participants achieved a minimum of 5 valid trials in each direction and could thus be included in the analysis. Conversely, the perturbation intensity was relatively low for PwS with less severe motor impairment, particularly when taking paretic steps in the anterior and anterolateral directions and for non-paretic steps. Hence, our experimental protocol may not have exposed the full range of stroke-related deficits, whereas the limitations of synergy analysis did not allow us to elucidate the coordination deficits in those who experienced the greatest difficulties stepping with the paretic leg. To solve these problems, it may be of interest to study whether the identified coordination patterns are consistent across different perturbation intensities. If this is the case, future studies may test participants at individualized intensities aligned to their reactive balance capacity.

### Clinical Implications

The present findings of deficient synergy structure and activation during reactive stepping complement and extend our insights in balance-related coordination impairments after supratentorial stroke. The profound difficulties in taking (postero)lateral steps, and the observed deficits in synergies that are preferentially recruited in these directions, suggest that training interventions may need to prioritize specific perturbation directions. The present findings demonstrate that stepping with either leg appears to be feasible for individuals with an FMA-LE score above 23 points. For those with a lower FMA-LE score and little paretic-leg stepping capacity in these directions, additional training of non-paretic cross stepping should be considered as an alternative.

Importantly, several studies have shown that reactive stepping capacity in PwS can be improved by perturbation-based training, yet the mechanisms of action remain elusive.^42-44^ The present results demonstrated stroke-related deficits not only in muscle synergy structures, but also in the degree of recruitment of several “normal” swing and stance leg synergies. In future studies on perturbation-based training, it would be of interest to identify whether previously reported improvements in reactive step quality, including posterior and lateral steps with the paretic leg,^43^ are related to optimization of pre-existing muscle coordination patterns, or whether some degree of behavioral restitution (i.e. return to “normal” muscle coordination patterns) may still be possible. Furthermore, it is of interest to investigate whether additional hip abductor strength training may enhance the efficacy of PBT for improving reactive step quality in the lateral directions, as has been shown in older adults combined training.^45^

## Supplemental Material

sj-docx-1-nnr-10.1177_15459683251369502 – Supplemental material for Deficient Muscle Coordination Patterns of Reactive Stepping Responses in People With Chronic Stroke Supplemental material, sj-docx-1-nnr-10.1177_15459683251369502 for Deficient Muscle Coordination Patterns of Reactive Stepping Responses in People With Chronic Stroke by Wouter Staring, Lotte van de Venis, Sarah Zandvliet, Digna de Kam, Teodoro Solis-Escalante, Alexander Geurts and Vivian Weerdesteyn in Neurorehabilitation and Neural Repair

sj-docx-2-nnr-10.1177_15459683251369502 – Supplemental material for Deficient Muscle Coordination Patterns of Reactive Stepping Responses in People With Chronic Stroke Supplemental material, sj-docx-2-nnr-10.1177_15459683251369502 for Deficient Muscle Coordination Patterns of Reactive Stepping Responses in People With Chronic Stroke by Wouter Staring, Lotte van de Venis, Sarah Zandvliet, Digna de Kam, Teodoro Solis-Escalante, Alexander Geurts and Vivian Weerdesteyn in Neurorehabilitation and Neural Repair

sj-docx-3-nnr-10.1177_15459683251369502 – Supplemental material for Deficient Muscle Coordination Patterns of Reactive Stepping Responses in People With Chronic Stroke Supplemental material, sj-docx-3-nnr-10.1177_15459683251369502 for Deficient Muscle Coordination Patterns of Reactive Stepping Responses in People With Chronic Stroke by Wouter Staring, Lotte van de Venis, Sarah Zandvliet, Digna de Kam, Teodoro Solis-Escalante, Alexander Geurts and Vivian Weerdesteyn in Neurorehabilitation and Neural Repair

## References

[1] Roelofs JMB Zandvliet SB Schut IM , et al Mild stroke, serious problems: limitations in balance and gait capacity and the impact on fall rate, and physical activity. Neurorehabil Neural Repair. 2023;37(11-12):786-798. doi:10.1177/15459683231207360 37877724 PMC10685695

[2] Forster A Young J . Incidence and consequences of falls due to stroke: a systematic inquiry. BMJ. 1995;311(6997):83-86. doi:10.1136/bmj.311.6997.83 7613406 PMC2550147

[3] Weerdesteyn V de Niet M van Duijnhoven HJ Geurts AC . Falls in individuals with stroke. J Rehabil Res Dev. 2008;45(8):1195-1213. 19235120

[4] de Kam D Geurts AC Weerdesteyn V Torres-Oviedo G . Direction-specific instability poststroke is associated with deficient motor modules for balance control. Neurorehabil Neural Repair. 2018;32(6-7):655-666. doi:10.1177/1545968318783884 29954244

[5] de Kam D Roelofs JMB Bruijnes A Geurts ACH Weerdesteyn V . The next step in understanding impaired reactive balance control in people with stroke: the role of defective early automatic postural responses. Neurorehabil Neural Repair. 2017;31(8):708-716. doi:10.1177/1545968317718267 28691582 PMC5714159

[6] Marigold DS Eng JJ . Altered timing of postural reflexes contributes to falling in persons with chronic stroke. Exp Brain Res. 2006;171(4):459-468. doi:10.1007/s00221-005-0293-6 16418855 PMC3226801

[7] Nashner LM . Adapting reflexes controlling the human posture. Exp Brain Res. 1976;26(1):59-72. doi:10.1007/BF00235249 964327

[8] Handelzalts S Steinberg-Henn F Levy S Shani G Soroker N Melzer I . Insufficient balance recovery following unannounced external perturbations in persons with stroke. Neurorehabil Neural Repair. 2019;33(9):730-739. doi:10.1177/1545968319862565 31315506

[9] Salot P Patel P Bhatt T . Reactive balance in individuals with chronic stroke: biomechanical factors related to perturbation-induced backward falling. Phys Ther. 2016;96(3):338-347. doi:10.2522/ptj.20150197 26206220

[10] Staring WHA van Duijnhoven HJR Roelofs JMB , et al Improvements in spatiotemporal outcomes, but not in recruitment of automatic postural responses, are correlated with improved step quality following perturbation-based balance training in chronic stroke. Front Sports Act Living. 2022;4:1008236. doi:10.3389/fspor.2022.1008236 36465583 PMC9714322

[11] Staring WHA Zandvliet S de Kam D Solis-Escalante T Geurts ACH Weerdesteyn V . Age-related changes in muscle coordination patterns of stepping responses to recover from loss of balance. Exp Gerontol. 2024;191:112424. doi:10.1016/j.exger.2024.112424 38604252

[12] Safavynia SA Torres-Oviedo G Ting LH . Muscle synergies: implications for clinical evaluation and rehabilitation of movement. Top Spinal Cord Inj Rehabil. 2011;17(1):16-24. doi:10.1310/sci1701-16 21796239 PMC3143193

[13] Barroso FO Torricelli D Moreno JC , et al Shared muscle synergies in human walking and cycling. J Neurophysiol. 2014;112(8):1984-1998. doi:10.1152/jn.00220.2014 25057144

[14] Chvatal SA Ting LH . Common muscle synergies for balance and walking. Front Comput Neurosci. 2013;7:48. doi:10.3389/fncom.2013.00048 23653605 PMC3641709

[15] Clark DJ Ting LH Zajac FE Neptune RR Kautz SA . Merging of healthy motor modules predicts reduced locomotor performance and muscle coordination complexity post-stroke. J Neurophysiol. 2010;103(2):844-857. doi:10.1152/jn.00825.2009 20007501 PMC2822696

[16] d’Avella A Bizzi E . Shared and specific muscle synergies in natural motor behaviors. Proc Natl Acad Sci USA. 2005;102(8):3076-3081. doi:10.1073/pnas.0500199102 15708969 PMC549495

[17] Routson RL Kautz SA Neptune RR . Modular organization across changing task demands in healthy and poststroke gait. Physiol Rep. 2014;2(6):e12055. doi:10.14814/phy2.12055 PMC4208640 24963035

[18] Van Criekinge T Vermeulen J Wagemans K , et al Lower limb muscle synergies during walking after stroke: a systematic review. Disabil Rehabil. 2020;42(20):2836-2845. doi:10.1080/09638288.2019.1578421 30905215

[19] Funato T Hattori N Yozu A , et al Muscle synergy analysis yields an efficient and physiologically relevant method of assessing stroke. Brain Commun. 2022;4(4):fcac200. doi:10.1093/braincomms/fcac200 PMC9374474 35974798

[20] Holden MK Gill KM Magliozzi MR Nathan J Piehl-Baker L . Clinical gait assessment in the neurologically impaired. Reliability and meaningfulness. Phys Ther. 1984;64(1):35-40. doi:10.1093/ptj/64.1.35 6691052

[21] Wilson B Cockburn J Halligan P . Development of a behavioral test of visuospatial neglect. Arch Phys Med Rehabil. 1987;68(2):98-102. 3813864

[22] Gladstone DJ Danells CJ Black SE . The fugl-meyer assessment of motor recovery after stroke: a critical review of its measurement properties. Neurorehabil Neural Repair. 2002;16(3):232-240. doi:10.1177/154596802401105171 12234086

[23] Collin C Wade D . Assessing motor impairment after stroke: a pilot reliability study. J Neurol Neurosurg Psychiatry. 1990;53(7):576-579. doi:10.1136/jnnp.53.7.576 2391521 PMC488133

[24] Verheyden G Nieuwboer A Mertin J Preger R Kiekens C De Weerdt W . The Trunk Impairment Scale: a new tool to measure motor impairment of the trunk after stroke. Clin Rehabil. 2004;18(3):326-334. doi:10.1191/0269215504cr733oa 15137564

[25] Franchignoni F Horak F Godi M Nardone A Giordano A . Using psychometric techniques to improve the Balance Evaluation Systems Test: the mini-BESTest. J Rehabil Med. 2010;42(4):323-331. doi:10.2340/16501977-0537 20461334 PMC3228839

[26] Martina IS van Koningsveld R Schmitz PI van der Meche FG van Doorn PA . Measuring vibration threshold with a graduated tuning fork in normal aging and in patients with polyneuropathy. European Inflammatory Neuropathy Cause and Treatment (INCAT) group. J Neurol Neurosurg Psychiatry. 1998;65(5):743-747. doi:10.1136/jnnp.65.5.743 9810949 PMC2170371

[27] Nonnekes J de Kam D Geurts AC Weerdesteyn V Bloem BR . Unraveling the mechanisms underlying postural instability in Parkinson’s disease using dynamic posturography. Expert Rev Neurother. 2013;13(12):1303-1308. doi:10.1586/14737175.2013.839231 24160682

[28] Hermens HJ Freriks B Disselhorst-Klug C Rau G . Development of recommendations for SEMG sensors and sensor placement procedures. J Electromyogr Kinesiol. 2000;10(5):361-374. doi:10.1016/s1050-6411(00)00027-4 11018445

[29] de Kam D Roelofs JMB Geurts ACH Weerdesteyn V . Body configuration at first stepping-foot contact predicts backward balance recovery capacity in people with chronic stroke. PLoS ONE. 2018;13(2):e0192961. doi:10.1371/journal.pone.0192961 PMC5823379 29470535

[30] Zandvoort CS van Dieen JH Dominici N Daffertshofer A . The human sensorimotor cortex fosters muscle synergies through cortico-synergy coherence. Neuroimage. 2019;199:30-37. doi:10.1016/j.neuroimage.2019.05.041 31121297

[31] Torres-Oviedo G Macpherson JM Ting LH . Muscle synergy organization is robust across a variety of postural perturbations. J Neurophysiol. 2006;96(3):1530-1546. doi:10.1152/jn.00810.2005 16775203

[32] Chvatal SA Ting LH . Voluntary and reactive recruitment of locomotor muscle synergies during perturbed walking. J Neurosci. 2012;32(35):12237-12250. doi:10.1523/JNEUROSCI.6344-11.2012 22933805 PMC3465667

[33] Pataky TC . Generalized n-dimensional biomechanical field analysis using statistical parametric mapping. J Biomech. 2010;43(10):1976-1982. doi:10.1016/j.jbiomech.2010.03.008 20434726

[34] Cheung VC Turolla A Agostini M , et al Muscle synergy patterns as physiological markers of motor cortical damage. Proc Natl Acad Sci USA. 2012;109(36):14652-14656. doi:10.1073/pnas.1212056109 22908288 PMC3437897

[35] Sanchez N Acosta AM Lopez-Rosado R Dewald JPA . Neural constraints affect the ability to generate hip abduction torques when combined with hip extension or ankle plantarflexion in chronic hemiparetic stroke. Front Neurol. 2018;9:564. doi:10.3389/fneur.2018.00564 30050495 PMC6050392

[36] Cruz TH Dhaher YY . Evidence of abnormal lower-limb torque coupling after stroke: an isometric study. Stroke. 2008;39(1):139-147. doi:10.1161/STROKEAHA.107.492413 18063824 PMC3641752

[37] Karatas M Cetin N Bayramoglu M Dilek A . Trunk muscle strength in relation to balance and functional disability in unihemispheric stroke patients. Am J Phys Med Rehabil. 2004;83(2):81-87. doi:10.1097/01.PHM.0000107486.99756.C7 14758293

[38] Cleland BT Madhavan S . Ipsilateral motor pathways to the lower limb after stroke: insights and opportunities. J Neurosci Res. 2021;99(6):1565-1578. doi:10.1002/jnr.24822 33665910 PMC8085051

[39] Gray VL Yang CL Fujimoto M McCombe Waller S Rogers MW . Stepping characteristics during externally induced lateral reactive and voluntary steps in chronic stroke. Gait Posture. 2019;71:198-204. doi:10.1016/j.gaitpost.2019.05.001 31078009 PMC6589388

[40] Pigman J Reisman DS Pohlig RT , et al Posterior fall-recovery training applied to individuals with chronic stroke: a single-group intervention study. Clin Biomech. 2021;82:105249. doi:10.1016/j.clinbiomech.2020.105249 PMC7940569 33421756

[41] Patel P Bhatt T . Modulation of reactive response to slip-like perturbations: effect of explicit cues on paretic versus non-paretic side stepping and fall-risk. Exp Brain Res. 2015;233(11):3047-3058. doi:10.1007/s00221-015-4367-9 26289480

[42] Mansfield A Aqui A Danells CJ , et al Does perturbation-based balance training prevent falls among individuals with chronic stroke? A randomised controlled trial. BMJ Open. 2018;8(8):e021510. doi:10.1136/bmjopen-2018-021510 PMC6104758 30121600

[43] van Duijnhoven HJR Roelofs JMB den Boer JJ , et al Perturbation-based balance training to improve step quality in the chronic phase after stroke: a proof-of-concept study. Front Neurol. 2018;9:980. doi:10.3389/fneur.2018.00980 30524360 PMC6261972

[44] Handelzalts S Kenner-Furman M Gray G Soroker N Shani G Melzer I . Effects of perturbation-based balance training in subacute persons with stroke: a randomized controlled trial. Neurorehabil Neural Repair. 2019;33(3):213-224. doi:10.1177/1545968319829453 30767613

[45] Rogers MW Creath RA Gray V , et al Comparison of lateral perturbation-induced step training and hip muscle strengthening exercise on balance and falls in community-dwelling older adults: a blinded randomized controlled trial. J Gerontol A Biol Sci Med Sci. 2021;76(9):e194-e202. doi:10.1093/gerona/glab017 PMC8361357 33491052

