# Supplementary material for: Deficient Muscle Coordination Patterns of Reactive Stepping Responses in People With Chronic Stroke
Source: Neurorehabil Neural Repair. 2025 Sep 15;39(12):1019–30. doi: 10.1177/15459683251369502 (PMC12686198; doi:10.1177/15459683251369502)
Supplement: sj-docx-1-nnr-10.1177_15459683251369502 – Supplemental material for Deficient Muscle Coordination Patterns of Reactive Stepping Responses in People With Chronic Stroke [file sj-docx-1-nnr-10.1177_15459683251369502.docx]

**Appendix 1. Linear mixed model (LMM) statistics across stepping characteristics**

|  | **Factor** | **F** | **P Value** |
| --- | --- | --- | --- |
| **Leg Angle** | group | 31..97 | **<0.01** |
|  | leg | 10.90 | **<0.01** |
|  | group * leg | 7 .80 | **<0.01** |
|  | direction * leg | 0 .09 | 0 .986 |
|  | direction * group * leg | 0 .01 | 1 .000 |
|  |  |  |  |
| **Step Duration** | group | 3.36 | .07 |
|  | leg | 4.09 | **.04** |
|  | group * leg | 16.2 | **<0.01** |
|  | direction * leg | 1.3 | .27 |
|  | direction * group * leg | 1.6 | .16 |
|  |  |  |  |
| **Step Length** | group | 19.0 | **<0.01** |
|  | leg | 1.3 | .24 |
|  | group * leg | 1.1 | .29 |
|  | direction * leg | .89 | .47 |
|  | direction * group * leg | .38 | .82 |
|  |  |  |  |
| **Step Onset** | group | 1.4 | .235 |
|  | leg | .26 | .61 |
|  | group * leg | .02 | .89 |
|  | direction * leg | .47 | .76 |
|  | direction * group * leg | .72 | .58 |

***Linear mixed model analysis per characteristic***

**Leg Angle**

|  | **direction** | **Leg** | **β estimate** | **P-value** | **Lower 95%** | **Upper 95%** |
| --- | --- | --- | --- | --- | --- | --- |
| **Paretic leg (reference)** | Ant |  | 13.9 |  | 12.9 | 14.8 |
|  |  | Non Paretic | 1.5 | **.01** | .4 | 2.8 |
|  |  | Dominant | 1.1 | .1 | -.2 | 2.4 |
|  | AntLat |  | 16.6 |  | 15.6 | 17.6 |
|  |  | Non Paretic | 1.6 | **0.1** | .35 | 2.8 |
|  |  | Dominant | .87 | .20 | -.49 | 2.2 |
|  | Lat |  | 16.5 |  | 15.5 | 17.6 |
|  |  | Non Paretic | 1.6 | **.01** | .34 | 2.9 |
|  |  | Dominant | 1.7 | **.02** | .29 | 3 |
|  | PostLat |  | 11.9 |  | 10.9 | 13.0 |
|  |  | Non Paretic | 1.7 | **.01** | .51 | 3.0 |
|  |  | Dominant | 3.7 | **<0.01** | 2.3 | 5.1 |
|  | Post |  | 8.9 |  | 7.9 | 9.9 |
|  |  | Non Paretic | 2.0 | **<0.01** | .82 | 3.3 |
|  |  | Dominant | 4.5 | **<0.01** | 3.2 | 5.9 |
|  |  |  |  |  |  |  |
| **Non dominant leg (reference)** | Ant |  | 15.02 |  | 13 .81 | 16 .22 |
|  |  | Dominant | -.02 | 0 .98 | -0.88 | 1.78 |
|  |  | Non paretic | 0 .45 | 0 .51 | -0 .88 | 1 .77 |
|  | AntLat |  | 17.5 |  | 16.3 | 18.7 |
|  |  | Dominant | -.01 | .98 | -1.4 | 1.3 |
|  |  | Non paretic | .70 | .30 | -.64 | 2.0 |
|  | Lat |  | 18.2 |  | 17.0 | 19.4 |
|  |  | Dominant | -.09 | .90 | -1.4 | 1.3 |
|  |  | Non paretic | -.03 | .97 | -1.4 | 1.4 |
|  | PostLat |  | 15.9 |  | 14.7 | 17.1 |
|  |  | Dominant | -.20 | .78 | -1.6 | 1.1 |
|  |  | Non paretic | -2.1 | ***<.01*** | -3.4 | -.77 |
|  | Post |  | 13.9 |  | 12.7 | 15.1 |
|  |  | Dominant | -.46 | .51 | -1.8 | .91 |
|  |  | Non paretic | -3 | ***<.01*** | -4.3 | -1.64 |

| **Reference leg** | **direction** | **Leg** | **β estimate** | **P-value** | **Lower 95%** | **Upper 95%** |
| --- | --- | --- | --- | --- | --- | --- |
| **Paretic leg (reference)** | Ant |  | 275 |  | 261 | 288 |
|  |  | Non Paretic | -41 | ***<.01*** | -30 | 9 |
|  |  | Dominant | -10 | .31 | -59 | -24 |
|  | AntLat |  | 207 |  | 192 | 221 |
|  |  | Non Paretic | -17 | .13 | -35 | 4 |
|  |  | Dominant | -15 | *.058* | -36 | .1 |
|  | Lat |  | 187 |  | 173 | 203 |
|  |  | Non Paretic | -12 | .2 | -30 | 6 |
|  |  | Dominant | -18 | .13 | -38 | 2 |
|  | PostLat |  | 213 |  | 197 | 227 |
|  |  | Non Paretic | -24 | .**01** | -43 | -6 |
|  |  | Dominant | -8 | .46 | -28 | 11 |
|  | Post |  | 269 |  | 255 | 283 |
|  |  | Non Paretic | 7 | <0.01 | -12 | 26 |
|  |  | Dominant | -28 | *.43* | -45 | -10 |
|  |  |  |  |  |  |  |
| **Non dominant leg (reference)** | Ant |  | 255 |  | 238 | 273 |
|  |  | Dominant | 8 | .33 | -10 | 28 |
|  |  | Non paretic | -22 | ***.02*** | -41 | -3 |
|  | AntLat |  | 206 |  | 189 | 224 |
|  |  | Dominant | -15 | .16 | -36 | 5.0 |
|  |  | Non paretic | -17 | .09 | -35 | 2.3 |
|  | Lat |  | 199 |  | 172 | 208 |
|  |  | Dominant | -17 | .09 | -41 | -.5 |
|  |  | Non paretic | -25 | **.01** | -34 | 4.8 |
|  | PostLat |  | 213 |  | 195 | 230 |
|  |  | Dominant | -9 | .44 | -28 | 11 |
|  |  | Non paretic | -25 | ***.01*** | -44 | -5 |
|  | Post |  | 281 |  | 263 | 298 |
|  |  | Dominant | -5 | .68 | -25 | 14 |
|  |  | Non paretic | -40 | ***<.01*** | -59 | -21 |

**Step Duration**

**Step Length**

| **Reference leg** | **direction** | **Leg** | **β estimate** | **P-value** | **Lower 95%** | **Upper 95%** |
| --- | --- | --- | --- | --- | --- | --- |
| **Paretic leg (reference)** | Ant |  | 411 |  | 385 | 437 |
|  |  | Non Paretic | -6 | .7 | -40 | 27 |
|  |  | Dominant | 12 | .51 | -25 | 50 |
|  | AntLat |  | 378 |  | 350 | 406 |
|  |  | Non Paretic | -28 | .114 | -63 | 6.8 |
|  |  | Dominant | 1.9 | .36 | -20 | 56 |
|  | Lat |  | 376 |  | 347 | 405 |
|  |  | Non Paretic | 15 | .42 | -21 | 51 |
|  |  | Dominant | 6.4 | .74 | -32 | 45 |
|  | PostLat |  | 365 |  | 336 | 395 |
|  |  | Non Paretic | 4 | .81 | -31 | 40 |
|  |  | Dominant | 38 | **.05** | -.4 | 77 |
|  | Post |  | 351 |  | 323 | 378 |
|  |  | Non Paretic | 20 | .253 | -14 | 54 |
|  |  | Dominant | 55 | ***<.01*** | 18 | 93 |
| **Non dominant leg (reference)** | Ant |  | 433 |  | 400 | 467 |
|  |  | Dominant | -10 | .6 | -48 | 8 |
|  |  | Non paretic | -29 | .13 | -66 | 28 |
|  | AntLat |  | 384 |  | 351 | 418 |
|  |  | Dominant | 11 | .56 | -27 | 50 |
|  |  | Non paretic | -34 | .07 | -72 | 3 |
|  | Lat |  | 413 |  | 379 | 448 |
|  |  | Dominant | -31 | .113 | -70 | 14.9 |
|  |  | Non paretic | -23 | .23 | -61 | 7.5 |
|  | PostLat |  | 425 |  | 391 | 458 |
|  |  | Dominant | -20 | .29 | -60 | 18 |
|  |  | Non paretic | -56 | ***<.01*** | -93 | -17 |
|  | Post |  | 442 |  | 408 | 476 |
|  |  | Dominant | 36 | .06 | -74 | 1.7 |
|  |  | Non paretic | -71 | ***<.01*** | -109 | -35 |
