# Supplementary material for: Deficient Muscle Coordination Patterns of Reactive Stepping Responses in People With Chronic Stroke
Source: Neurorehabil Neural Repair. 2025 Sep 15;39(12):1019–30. doi: 10.1177/15459683251369502 (PMC12686198; doi:10.1177/15459683251369502)
Supplement: sj-docx-2-nnr-10.1177_15459683251369502 – Supplemental material for Deficient Muscle Coordination Patterns of Reactive Stepping Responses in People With Chronic Stroke [file sj-docx-2-nnr-10.1177_15459683251369502.docx]

**Appendix 2.1 Flow of participants in the ROADS study**

**
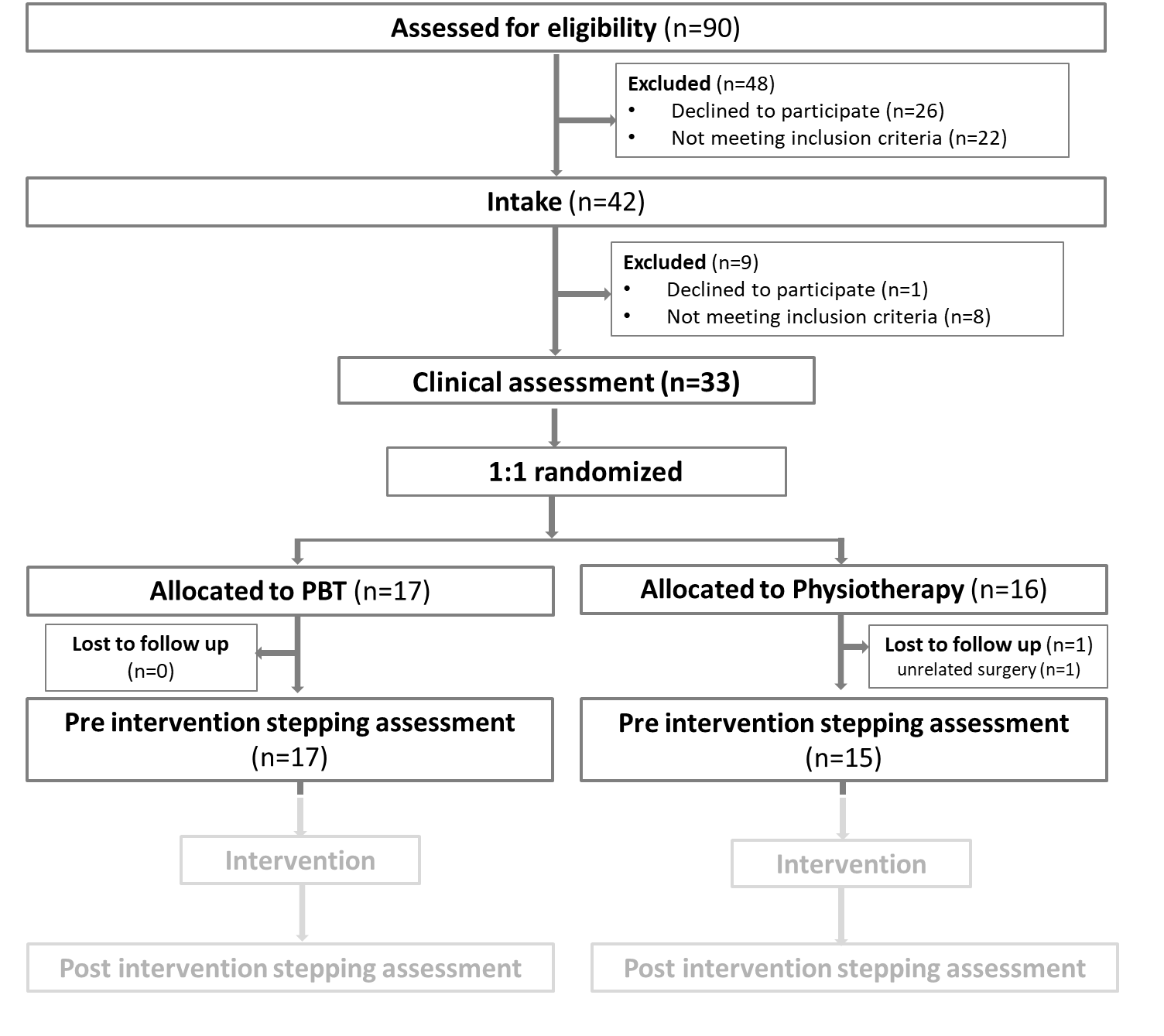
**

**Appendix 2.2 Experimental protocol: Platform movement & sequence order**


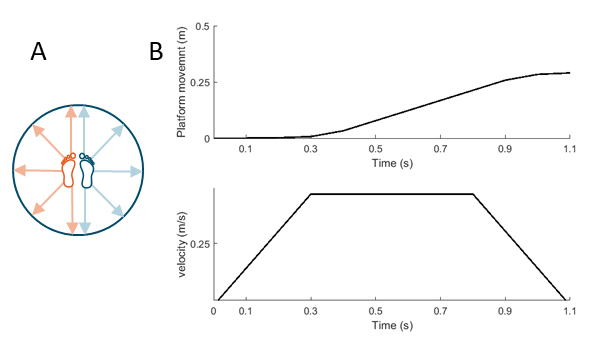


Figure A represents the perturbations directions (i.e., stepping direction) for each leg, whereas fig b shows the waveform of platform displacement (upper) and velocity (lower).

**Sequence order of perturbations and mandatory break**

The protocol consisted of 4 sequences per stepping leg. We generated a total of 8 random sequences (A-D for the Right leg) and E-H for the left leg. In addition, we randomly assigned which would be starting leg (left or right or paretic or non-paretic). At the start of the experiment we would select a sequence order (A-D & E-H) for each leg. After the mandatory break we counterbalance the “starting leg”.

*Flow diagram of perturbation series for a participant with a starting “paretic right leg”*

**
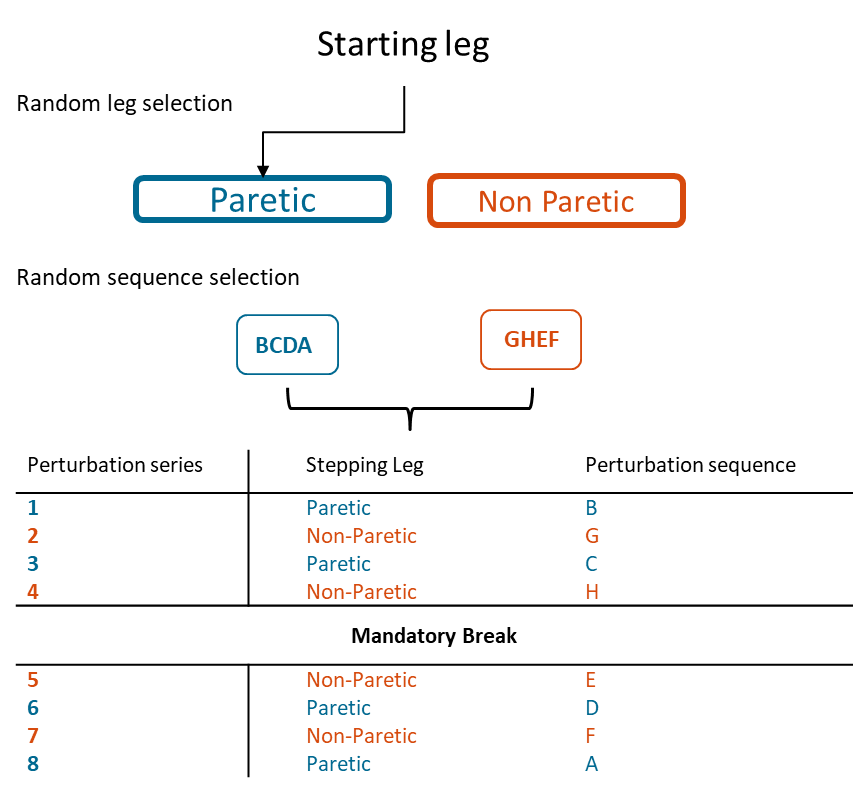
**
