# Supplementary material for: Deficient Muscle Coordination Patterns of Reactive Stepping Responses in People With Chronic Stroke
Source: Neurorehabil Neural Repair. 2025 Sep 15;39(12):1019–30. doi: 10.1177/15459683251369502 (PMC12686198; doi:10.1177/15459683251369502)
Supplement: sj-docx-3-nnr-10.1177_15459683251369502 – Supplemental material for Deficient Muscle Coordination Patterns of Reactive Stepping Responses in People With Chronic Stroke [file sj-docx-3-nnr-10.1177_15459683251369502.docx]

|  | Item No. | Recommendation | Page  No. | Relevant text from manuscript |
| --- | --- | --- | --- | --- |
| **Title and abstract** | 1 | (*a*) Indicate the study’s design with a commonly used term in the title or the abstract | 1 | Title*: Deficient muscle coordination patterns of reactive stepping responses in people with chronic stroke* |
|  |  | (*b*) Provide in the abstract an informative and balanced summary of what was done and what was found | 2 | Provided in abstract on Page 2 |
| **Introduction** | | | | |
| Background/rationale | 2 | Explain the scientific background and rationale for the investigation being reported | 4 | Provided in introduction. page 4, paragraph 2,3 |
| Objectives | 3 | State specific objectives, including any prespecified hypotheses | 5 | Provided on page 5 paragraph 2: *“In this study, we aimed to identify stroke-specific deficits in muscle coordination patterns of reactive stepping. We used muscle synergy analysis to identify deficits across multidirectional stepping responses in paretic and non-paretic swing and stance legs.”* |
| **Methods** | | | | |
| Study design | 4 | Present key elements of study design early in the paper | 6,7 | Provided in method section page 6,7, *“Experimental procedure & Reactive stepping assessment”* |
| Setting | 5 | Describe the setting, locations, and relevant dates, including periods of recruitment, exposure, follow-up, and data collection | 6,7 | Provided in method section page 6,7 ,*“Experimental procedure & Reactive stepping assessment”* |
| Participants | 6 | *Case-control study*—Give the eligibility criteria, and the sources and methods of case ascertainment and control selection. Give the rationale for the choice of cases and controls | 6 | Provided in method section page 6, *“Participants”* |
|  |  | *Case-control study*—For matched studies, give matching criteria and the number of controls per case | 6 | Provided in method section Page 6, *“Participants”* |
| Variables | 7 | Clearly define all outcomes, exposures, predictors, potential confounders, and effect modifiers. Give diagnostic criteria, if applicable | 8,9 | Provided in method section page 8, 9, “*Spatiotemporal characteristics and step quality & Muscle synergy analysis”* |
| Data sources/ measurement | 8* | For each variable of interest, give sources of data and details of methods of assessment (measurement). Describe comparability of assessment methods if there is more than one group | 7,8 | Provided in method section page 7, “*Reactive stepping assessment* “  page 8 , *“Data processing & analysis”* |
| Bias | 9 | Describe any efforts to address potential sources of bias | 7 | Randomization procedures have been described on page 7 , “*Reactive stepping assessment* “ |
| Study size | 10 | Explain how the study size was arrived at | 6 | Provided in method section page 6, *“Participants”* |

| Quantitative variables | 11 | Explain how quantitative variables were handled in the analyses. If applicable, describe which groupings were chosen and why |  | n/a- |
| --- | --- | --- | --- | --- |
| Statistical methods | 12 | (*a*) Describe all statistical methods, including those used to control for confounding | 10 | Provided in method section page 10, *“Statistical analysis”* |
|  |  | (*b*) Describe any methods used to examine subgroups and interactions | 10 | Provided in method section page 10, Statistical analysis: “*To identify differences in step characteristics between PwS and healthy controls, we performed linear mixed model (LMM) analyses with fixed factors group (healthy vs. stroke), leg (paretic or dominant vs. non-paretic or non-dominant), stepping direction (5 levels) and the respective interactions between these parameters”* |
|  |  | *Case-control study*—If applicable, explain how matching of cases and controls was addressed |  | Provided in method section page 6, *“Participants”* |
|  |  | (*e*) Describe any sensitivity analyses |  | - |
| **Results** | | | | |
| Participants | 13* | (a) Report numbers of individuals at each stage of study—eg numbers potentially eligible, examined for eligibility, confirmed eligible, included in the study, completing follow-up, and analysed | 11 | Provided in results, page 11, paragraph 1 & Appendix 2 prisma flow chart |
|  |  | (b) Give reasons for non-participation at each stage | 12 |  |
| Descriptive data | 14* | (c) Consider use of a flow diagram |  | Appendix 2 |
|  |  | (a) Give characteristics of study participants (eg demographic, clinical, social) and information on exposures and potential confounders | 11 | Provided in results, page 11, “*Table 1 participant characteristics”* |
|  |  | (b) Indicate number of participants with missing data for each variable of interest | 12 | Provided in results, page 12, paragraph 1 |
| Outcome data | 15* |  |  |  |
|  |  | *Case-control study—*Report numbers in each exposure category, or summary measures of exposure | *12,14* | Provided in results, page 12, paragraph 1 describes total number of steps  Page 14, paragraph Muscle coordination patterns describes number of participants for synergy analysis |
| Main results | 16 | (*a*) Give unadjusted estimates and, if applicable, confounder-adjusted estimates and their precision (eg, 95% confidence interval). Make clear which confounders were adjusted for and why they were included | 13 | Provided in results, page 13, paragraph Step quality and spatiotemporal characteristics  A full overview of the statistics can be found in Appendix 1 |
|  |  | (*b*) Report category boundaries when continuous variables were categorized | 16,17 | Provided in results, page 16,17, paragraph *“Muscle synergy activation patterns”* |
|  |  | (*c*) If relevant, consider translating estimates of relative risk into absolute risk for a meaningful time period |  |  |

| Other analyses | 17 | Report other analyses done—eg analyses of subgroups and interactions, and sensitivity analyses | 15 | Provided in results, page 15 paragraph 1 |
| --- | --- | --- | --- | --- |
| Discussion | | | | |
| Key results | 18 |  |  |  |
|  |  | Summarise key results with reference to study objectives | 18 | Provided in discussion, page 18, paragraph 1 |
| **Limitations** | | | | |
| Interpretation | 20 | Give a cautious overall interpretation of results considering objectives, limitations, multiplicity of analyses, results from similar studies, and other relevant evidence | 22 | Provided in discussion, page 22 , *“Clinical implications”* |
| Generalisability | 21 | Discuss the generalisability (external validity) of the study results | 21,22 | Provided in discussion, page 21- 22 “*Limitations and implications”*& *“Clinical implications”* |
| **Other information** | | | | |
| Funding | 22 | Give the source of funding and the role of the funders for the present study and, if applicable, for the original study on which the present article is based | 1 | *This work was funded by a Netherlands Organization for Scientific Research (NWO) VIDI grant awarded to VW (No.9171736), Project- Roads to recovery* |
| Other analyses | 17 | Report other analyses done—eg analyses of subgroups and interactions, and sensitivity analyses | 15 | Provided in results, page 15 paragraph 1 |
| Discussion  Key results | 18 |  |  |  |
|  |  | Summarise key results with reference to study objectives | 18 | Provided in discussion, page 18, paragraph 1 |
| Limitations | 19 | Discuss limitations of the study, taking into account sources of potential bias or imprecision. Discuss both direction and magnitude of any potential bias | 21 | Provided in discussion, page 21, “*Limitations and implications”* |
| Interpretation | 20 | Give a cautious overall interpretation of results considering objectives, limitations, multiplicity of analyses, results from similar studies, and other relevant evidence | 22 | Provided in discussion, page 22 , *“Clinical implications”* |
| Generalisability | 21 | Discuss the generalisability (external validity) of the study results | 21,22 | Provided in discussion, page 21- 22 “*Limitations and implications”*& *“Clinical implications”* |
| Other information |  |  |  |  |
| Funding | 22 | Give the source of funding and the role of the funders for the present study and, if applicable, for the original study on which the present article is based | 1 | *This work was funded by a Netherlands Organization for Scientific Research (NWO) VIDI grant awarded to VW (No.9171736), Project- Roads to recovery* |

*Give information separately for cases and controls in case-control studies and, if applicable, for exposed and unexposed groups in cohort and cross-sectional studies.

**Note:** An Explanation and Elaboration article discusses each checklist item and gives methodological background and published examples of transparent reporting. The STROBE checklist is best used in conjunction with this article (freely available on the Web sites of PLoS Medicine at http://www.plosmedicine.org/, Annals of Internal Medicine at http://www.annals.org/, and Epidemiology at http://www.epidem.com/). Information on the STROBE Initiative is available at www.strobe-statement.org.
